# Epilepsy in hypothalamic hamartomas: semiology spectrum and predictor analyses of 78 patients

**DOI:** 10.1002/acn3.51827

**Published:** 2023-06-27

**Authors:** Xiu Wang, Chang Liu, Zhong Zheng, Wenhan Hu, Chao Zhang, Xiaoli Yang, Xiaoqiu Shao, Jian‐Guo Zhang, Kai Zhang

**Affiliations:** ^1^ Department of Neurosurgery Beijing Tian Tan Hospital, Capital Medical University Beijing 100070 China; ^2^ Epilepsy Center Medical Alliance of Beijing Tian Tan Hospital, Peking University First Hospital Fengtai Hospital Beijing 100071 China; ^3^ Stereotactic and Functional Neurosurgery Laboratory Beijing Neurosurgical Institute, Capital Medical University Beijing 100070 China; ^4^ Department of Neurology Beijing Tian Tan Hospital, Capital Medical University Beijing 100070 China; ^5^ Beijing Key Laboratory of Neurostimulation Beijing 100070 China

## Abstract

**Objective:**

To assess seizure semiology and disease evolution in a large number of hypothalamic hamartoma (HH) patients.

**Methods:**

Seizure semiology and associated medical records for 78 patients with HH‐related epilepsy were retrospectively reviewed. Potential predictors of seizure types were assessed through univariate and binary logistic regression analyses.

**Results:**

57 (73.1%) patients presented with gelastic seizures at the onset of epilepsy, of whole 39 (68.4%) experienced additional seizure types with a mean latency interval of 4.59 years. Automatism, version, and sGTCs were increasingly common with disease evolution. The intraventricular size of HH was significantly negatively correlated with the disease evolution interval (*r* = −0.445, *p* = 0.009). A significantly higher rate of patients with automatism in the DF‐II group relative to the DF‐III group was found in both *χ* ^2^ (*X* = 6.07, *p* = 0.014) and logistic regression analyses (*B* = 3.196, *p* = 0.020).

**Interpretation:**

Gelastic seizures are the most common initial seizure type in HH patients, but variable semiologies occur with disease evolution. The intraventricular HH lesion size is an important determinant of epilepsy evolution. DF‐II HH lesions contribute to a higher chance of automatism evolution. The present study furthers our understanding of the dynamic organization of the seizure network affected by HH.

## Introduction

Hypothalamic hamartomas (HH) are benign brain lesions comprised of ectopic glial and neural tissue that are an important cause of treatment‐resistant epilepsy, especially in pediatric patients.^1^ Most HH patients experience distinctive gelastic seizures (GS) during the first year of life. Epilepsy cases typically exhibit a progressive natural history,^2^ and HH patients can exhibit a distinct epilepsy syndrome with seizure semiology that varies in terms of both severity and evolution.^3^ With time, other seizure semiologies may develop in these patients including automatism, dacrystic, version, spasms, dialeptic seizures with behavioral arrest, and focal to bilateral tonic–clonic seizures (often called secondarily generalized tonic–clonic seizures, sGTCs).^4^, ^5^ Conducting detailed seizure semiology analyses in patients exhibiting a range of ages and disease durations thus has the potential to provide more detailed insight into epileptogenesis and the evolution of HH‐related epilepsy.^5^ Various seizure manifestations may be associated with anatomical or functional connections between the hypothalamus and the frontal lobes, thalamus, and limbic circuitry.^3^ No definitive predictors of disease variation have been established in cases of HH‐related epilepsy, although there is prior evidence suggesting that seizure semiology and disease evolution may be impacted by lesion size,^6^ location,^7^ and the invasion of hypothalamic structures.^7^ These studies have, however, been relatively small, thus limiting their reliability. To further probe this important topic, a series of HH patients with epilepsy were collected to characterize their clinical and anatomical features in the present study. In addition, the clinical spectrum of HH‐related seizure semiology was evaluated with the goal of determining whether HH neuroanatomical features can impact the evolution of seizure semiology.

## Methods

### Patient selection

In total, 78 patients that were evaluated at the epilepsy centers of Beijing Fengtai Hospital and Beijing Tiantan Hospital from August 2015–April 2022 were retrospectively analyzed for this study. HH was usually characterized by a T1 signal similar to that of the cortex and increased T2 signal strength in magnetic resonance imaging (MRI).^7^ The diagnosis for each patient was made in a multidisciplinary presurgical evaluation conference including neurosurgeon, neurologist, electrophysiologist, neuroradiologist, and neuropsychiatrist. Cases were excluded if they met any of the following criteria: (1) no confirmed disease evolution information in the epilepsy history recordings; (2) any history of prior neurosurgical or radiosurgical treatment; (3) a lack of any high‐resolution MRI data for anatomical analyses; or (4) patients with other brain abnormalities detectable on MRI analysis. This cohort was comprised of HH patients referred for epilepsy treatment, and there were thus no HH patients without seizures included in this study. The Institutional Review Board of Beijing Tiantan Hospital approved the present study and all patients provided written informed consent.

### Seizure semiology analyses

Disease evolution was assessed based on detailed clinical recordings. All patients underwent video‐electroencephalography (VEEG) monitoring and the recording of habitual seizure events, with seizure types being determined based on a combination of this monitoring and clinical interviews. The following semiology components included in this study were selected based on our clinical experience and prior semiology reports^5^, ^8^: gelastic seizures (GS), gelastic seizures plus (GS+), dacrystic seizures, vegetative signs, manual or oral automatism, version, tonic or clonic seizures, spasms, sGTCs, eyelid blinking, staring or behavioral arrest. As sGTCs incidence is rare, this semiologic element was primarily confirmed through clinical interviews, whereas all other elements had to be observed during VEEG analyses or in videos provided by patients or their caregivers. GS+ was defined as a combination of GS and other semiologic elements in an ictal event, including partial or sGTCs.^8^ Vegetative signs included facial flushing and an elevated heart rate. A neurologist (X.Q.S) and a neurosurgeon (W.H.H) specializing in epilepsy independently analyzed seizure semiology, with discrepancies being resolved through discussion with a senior neurosurgeon (K.Z).

### HH anatomical feature analyses

High‐resolution whole brain MRI scanning was performed for each patient with a 3T Siemens Verio scanner (Siemens Medical System, NJ, USA) via a 3D T1 sagittal magnetization prepared rapid gradient echo sequence (MPRAGE, TR/TE 1900/2.53, TI 900, matrix 256 × 256, 1.0 mm thickness), T2 axial (TR/TE 7030/110, matrix 256 × 320, 3 mm thickness), FLAIR axial (TR/TE 8000/94, TI 2371.5, matrix 424 × 512, 3 mm thickness), FLAIR sagittal (TR/TE 8000/96, TI 2371.2, matrix 236 × 256, 3 mm thickness), FLAIR coronal (TR/TE 8000/96, TI 2371.2, matrix 408 C 512, 3 mm thickness), T2 STIR axial (TR/TE6520/21, matrix 256 × 256 × 256 mm, 2 mm thickness), T2 STIR sagittal (TR/TE6520/21, matrix 256 × 256 × 256 mm, 2 mm thickness), and T2 STIR coronal (TR/TE6520/21, matrix 256 × 256 × 256 mm, 2 mm thickness) sequences.

**Figure 1:**
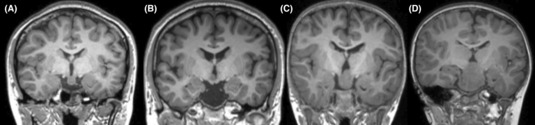
Delalande and Fohlen (DF) classifications for hypothalamic hamartomas. (A), (B), (C) and (D) refer to DF classifications types I, II, III and IV, respectively.

The Delalande and Fohlen (DF) classification system was then used to characterize these HH lesions^9^ (Fig. 1). Type 1 lesions exhibited a horizontal orientation and were lateralized to one side in some cases, typically with an extraventricular location. Type II lesions exhibited a vertical orientation and an intraventricular location. Type III lesions were a combination of types I and II, while Type IV lesions were giant hamartomas. A region of interest (ROI) was defined based on the HH borderline, and the ROI volume was calculated with 3D‐slicer. For type II DF (DF‐II) HH cases, the intraventricular portion of the lesion was also selected as part of the ROI. However, as the hypothalamus borderline was distorted and the precise intraventricular component of the lesion could not be reliably determined, a uniform set of criteria were used to select these ROIs. Specifically, on axial images parallel to the anterior commissure (AC)‐posterior commissure (PC) plane, the intraventricular portion of DF‐II lesions was defined as the lesion above the superior border of the optic tract at the level of AC. Two epilepsy neurosurgeons (W.H.H and Z.Z) experienced in HH ablation surgery procedures independently performed ROI selection. Final ROI volumes were the average of the volumes for ROIs established by these investigators.

Three primary zones along the mediolateral axis from the midline of the third ventricle were considered: the thin periventricular, medial, and lateral zones, with the latter two being separated by a sagittal plane passing through the anterior pillar of the fornix.^10^ To analyze the extent of invasion, the maximum lateral depth of the HH lesion (lateral to the line between the mid‐points of the fornix and the mamillothalamic tract in the axial plane parallel to the AC–PC plane) was analyzed. HH anatomical dimensions were assessed by measuring the corresponding anterior and posterior dimensions in relation to the central point of the mammillary body (MB)^8^ (Fig. S1). In cases of MB dislocation, the contralateral MB served as a reference. In rare cases of bilateral MB dislocation, these cases were discarded (*N* = 5). MB involvement was considered evident when the interruption of the halo of T1 hyperenhancement surrounding these structures in contact with the HH mass was evident^7^ (Fig. S2).

### Statistical analyses

Semiologic elements for each patient were analyzed, with the semiology frequency being determined based on the number of patients with each element as a fraction of the total patient population. Given that DF‐I cases exhibit atypical clinical presentations and DF‐IV cases are rare, analyses of the relationship between anatomical characteristics and epilepsy evolution were conducted for patients in groups DF‐II and DF‐III. Evolution analyses were conducted for patients that exhibited GS as the initial seizure event and additional seizure types during disease evolution. The disease evolution period (DEP) was calculated as the interval between the onset of gelastic and non‐gelastic seizures. Categorical data were compared with Fisher's exact test, while continuous data were compared with *t*‐tests and ANOVAs. *p* < 0.05 was the significance level. Predictors of ictal seizure semiology were identified through binary logistic regression analysis.

## Results

### Patient demographic characteristics and epilepsy history

This study included 78 patients (male: female = 53:25) with a mean age of 3.8 ± 5.4 years (range: 0.1–29.0 years) at seizure onset and a mean age of 9.9 ± 8.7 years (range: 1.7–38.0 years) at time of VEEG evaluation. The mean disease duration for this patient cohort was 6.1 ± 6.5 years (range: 0.1–30 years). Precocious puberty was observed in 16 patients. Of these patients, 15, 30, 28, and 5 were classified into the DF‐I, DF‐II, DF‐III, and DF‐IV groups, respectively, with corresponding mean ages of seizure onset of 12.12 ± 7.17, 2.29 ± 2.34, 1.54 ± 1.53, and 0.75 ± 0.78 years old. Disease onset in group DF‐I was significantly later than in the three other groups (*p* < 0.001). GS were present at the onset of epilepsy in 57 (73.1%) patients, with a mean age of 2.10 ± 2.46 years (range: 0.1–11 years) at seizure onset. With respect to the percentage of patients in these different groups presenting with GS at the time of initial epilepsy onset, significantly more patients in groups DF‐II (26/30, 86.7%), III (24/28, 85.7%), and IV (4/5, 80%) experienced GS during their first ictal event relative to group DF‐I (3/15, 20%) (*χ* ^2^ = 23.21, *p* < 0.001). Additionally, 39 of these patients (68.4%) exhibited other seizure types over the course of epilepsy evolution with a mean latency interval of 4.35 ± 3.89 years (range 0.1–13 years) and mean onset age of 7.22 ± 5.07 years (range: 0.9–21 years) (Table 1).

**Table 1:** General features and lesion volume in patients with hypothalamic hamartoma according to Delalande and Fohlen classifications.

F/M(N)	DF‐I	DF‐II	DF‐III	DF‐IV	Total	*p*
	8/7	7/23	9/19	1/4	25/53	ns
Age at onset (years, mean ± SD)	12.12 ± 7.17	2.29 ± 2.34	1.54 ± 1.53	0.75 ± 0.78	3.81 ± 5.39	*p* < 0.001
Age at evaluation (years, mean ± SD)	17.95 ± 9.47	10.38 ± 8.11	5.67 ± 6.00	7.05 ± 5.84	9.93 ± 8.67	*p* < 0.001
Disease duration (years, mean ± SD)	5.83 ± 6.37	8.09 ± 7.02	4.13 ± 5.66	6.29 ± 5.92	6.12 ± 6.47	ns
Precocious puberty (*N*)	2	2	8	4	16	*p* = 0.002
GS (*N*, onset age)	4 (11.00 ± 12.43)	26 (2.24 ± 2.24)	25 (1.36 ± 1.37)	4 (0.90 ± 0.82)	59 (2.37 ± 4.11)	*p* < 0.001
Non‐GS (*N*, onset age)	15 (12.87 ± 7.46)	21 (7.75 ± 4.89)	20 (3.96 ± 3.29)	4 (5.99 ± 6.46)	60 (7.65 ± 6.22)	*p* < 0.001
Total volume of HH (mm^3^)	1286.00 ± 1291.91	270.00 ± 142.49	2057.86 ± 1504.84	15040.00 ± 5282.38	2053.97 ± 3854.87	*p* < 0.001
Volume of intraventricular HH (mm^3^)	NA	270.00 ± 142.49	786.07 ± 566.96	NA	519.14 ± 479.84	*p* < 0.001

DF, Delalande and Fohlen classifications; F, female; M, male; GS, gelastic seizures; HH, hypothalamic hamartoma.

### Seizure semiology

With respect to seizure semiology in VEEG analyses, 74.36% (58/78) and 28.21% (22/78) of patients exhibited GS and GS+ semiology, respectively, while 7.69% (6/78) exhibited dacrystic seizures. Vegetative signs such as facial flush, elevated heart rate, and elevated respiratory rate often occur during the early phases of GS or GS+ and were observed in 33.33% (23/69) of these patients. GS frequency in group DF‐I (4/15, 26%) was lower than that in groups II (25/30, 83.33%), III (25/28, 89.29%), and IV (4/5, 80%).

Ictal version occurred in 23 patients (29.49%), with similar prevalence rates in the DF‐I (5/15, 33.33%), II (10/30, 33.33%), III (7/28, 25.00%), and IV groups (1/5, 20%), exhibiting lateralization in 18 patients (78.3%). Automatism (29.49%), including manual and oral automatism, was also common in HH‐related epilepsy patients. Seizures with automatism were more common in the DF‐I (6/15, 40%) and II (12/30, 40%) groups relative to groups III (4/28, 14.29%) and IV (1/5, 20%). Of these patients, 32.05% exhibited sGTCs, with these rates being highest in group DF‐I (53.3%).

**Figure 2:**
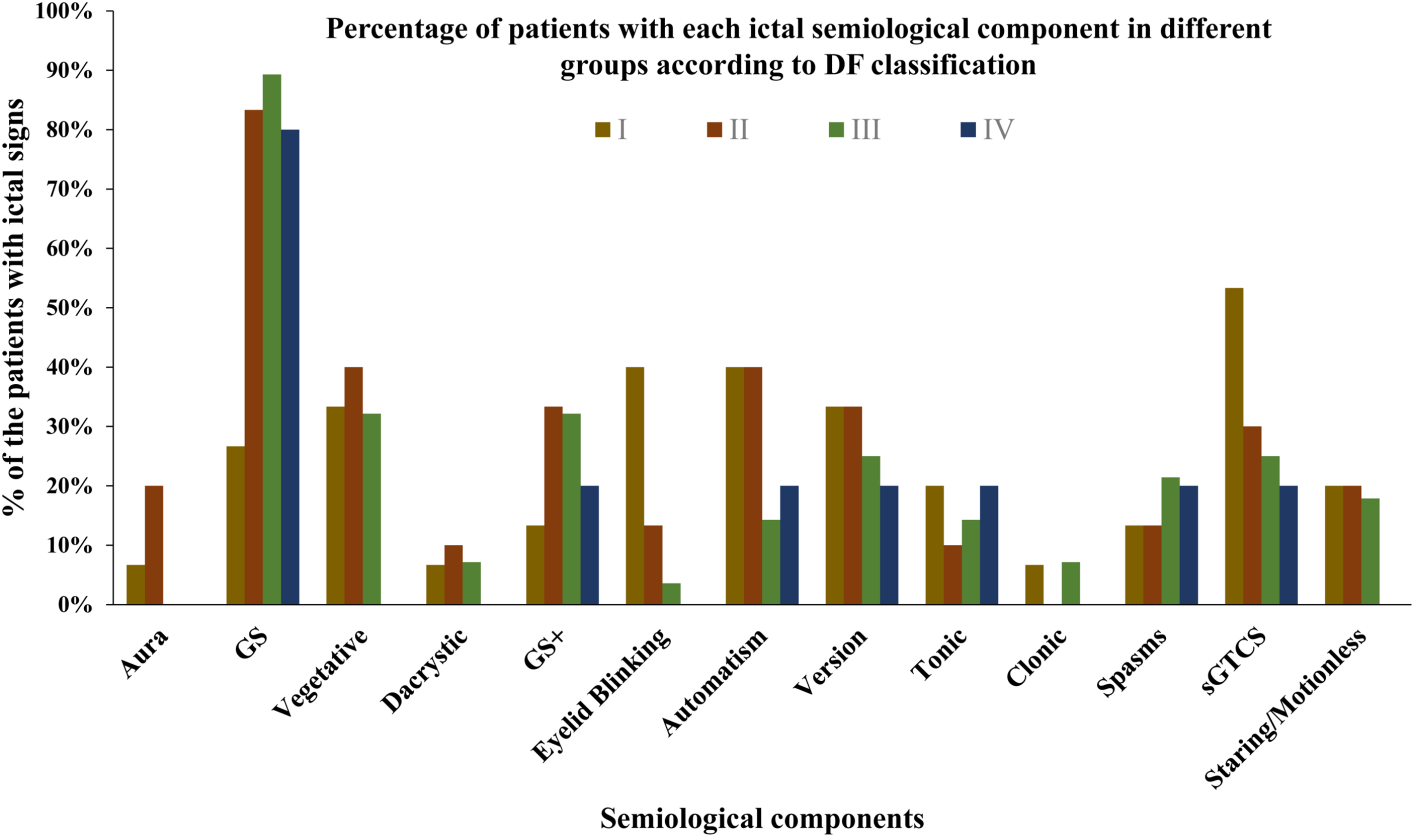
The percentages of patients with the indicated semiological components based on DF classification. GS, gelastic seizures; GS+, gelastic seizures plus.

Tonic seizures, including those involving the unilateral or bilateral extremities or axial tonic seizures, affected 14.10% of these patients. The prevalence rates for other seizure signs including blinking, clonic, spasms, and behavioral arrest were 14.10%, 3.85%, 16.67%, and 17.95%, respectively. For further details regarding the rates of these semiologic signs in each of these DF subgroups, see Fig. 2.

### Disease evolution and seizure features

**Figure 3:**
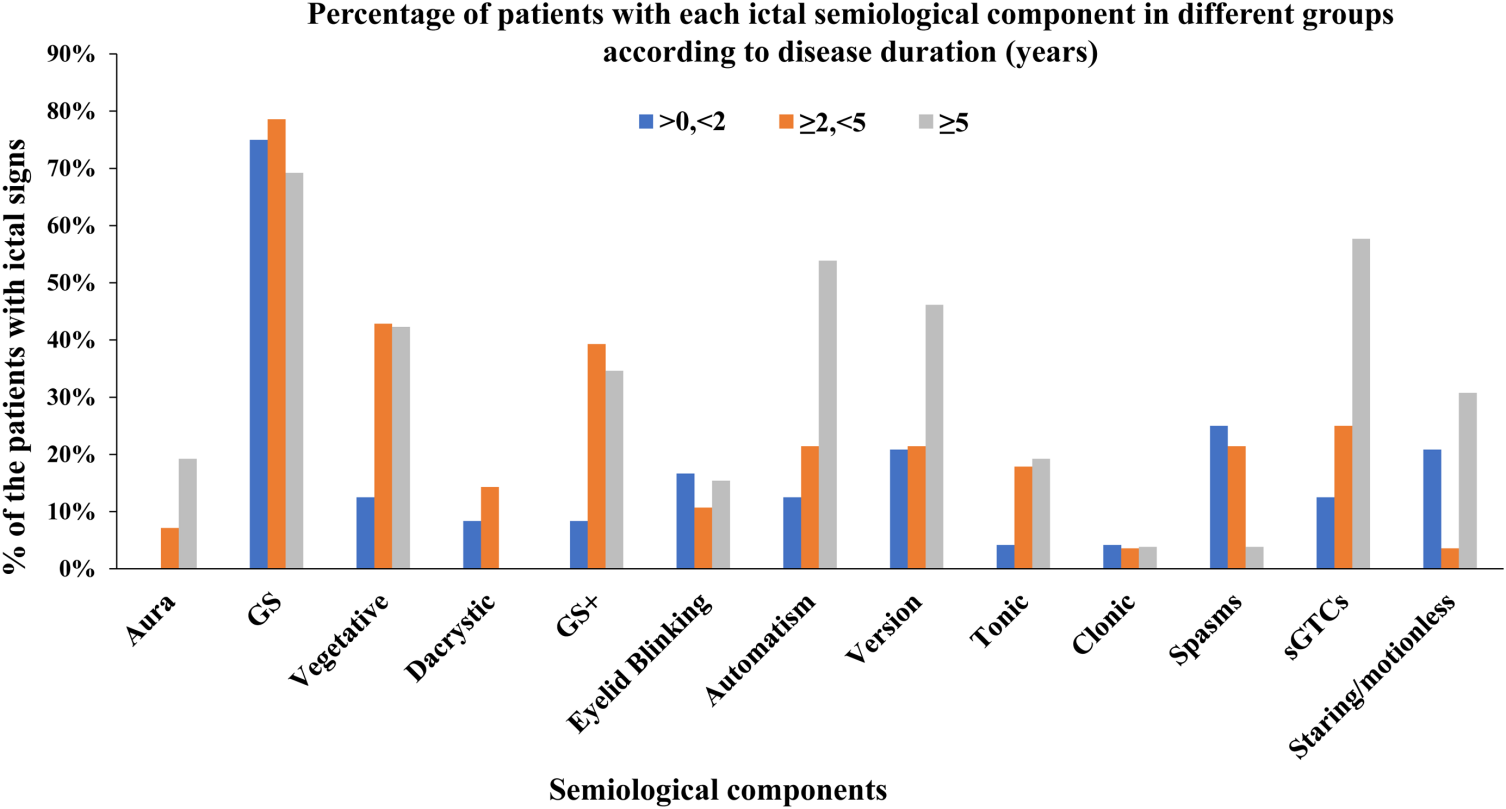
The percentages of patients with the indicated semiological components based on disease duration in years. GS, gelastic seizures; GS+, gelastic seizures plus.

When analyzing disease duration, semiologic changes were grouped into short (<2 years), intermediate (2–5 years), and long disease duration (≥5 years) groups. GS percentages did not change significantly over the course of disease evolution, whereas the frequencies of semiologic signs, including autonomic signs (12.5%, 42.9%, and 42.3%), GS+ (8.3%, 39.3%, and 34.6%), automatism (12.5%, 21.4%, and 53.9%), ictal version (20.8%, 21.4%, and 46.2%), and sGTCs (12.5%, 25.0%, and 57.7%) rose with the prolongation of disease duration (Fig. 3).

**Figure 4:**
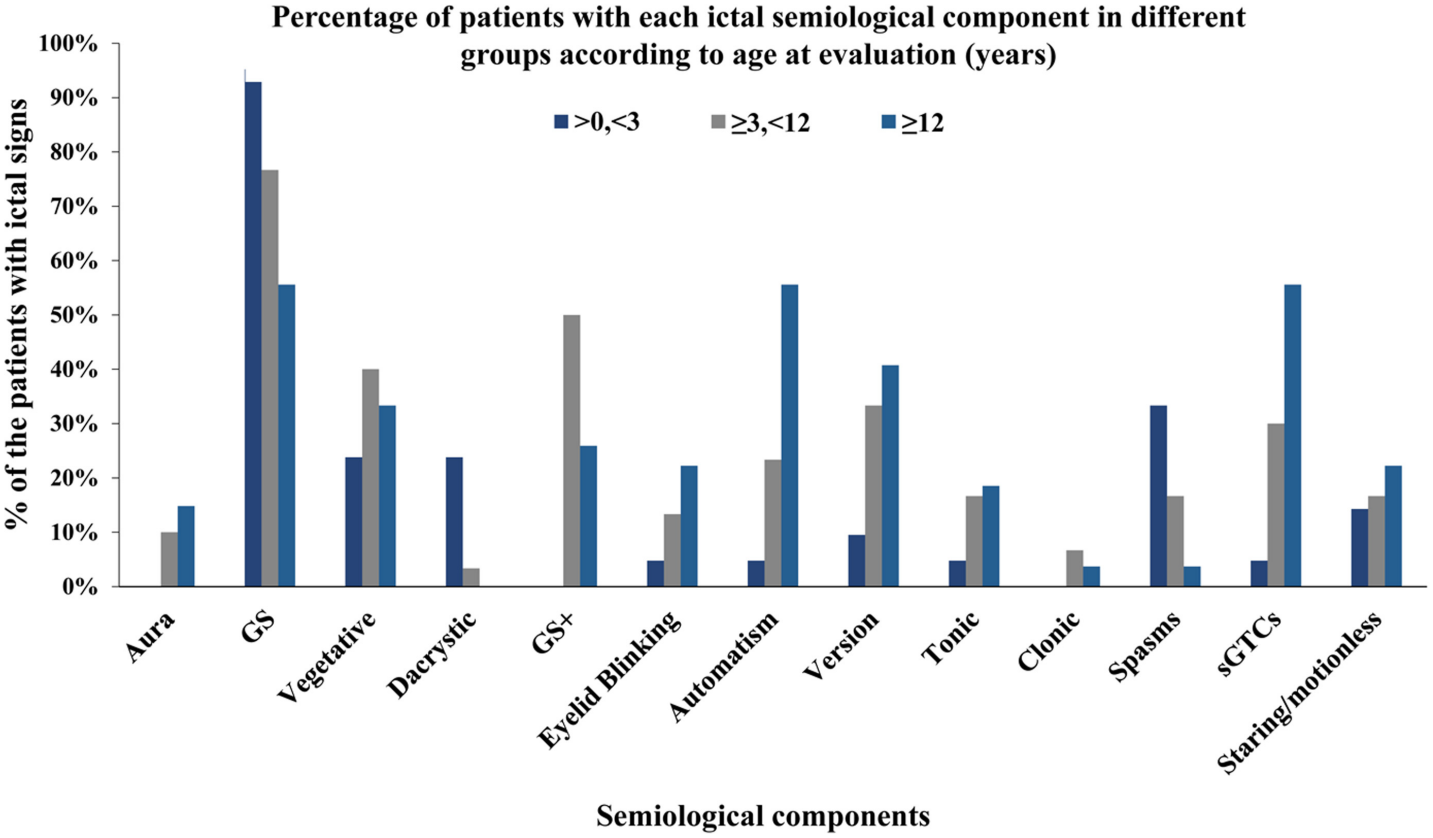
The percentages of patients with the indicated semiological components based on age at evaluation in years. GS, gelastic seizures; GS+, gelastic seizures plus.

With respect to the age of VEEG evaluation for semiology changes, subgroups included infant (<3 years), children (3–12 years), and adolescent‐adult (≥12 years) groups. Automatism (4.8%, 23.3%, and 55.6%), ictal version (9.5%, 33.3%, and 40.7%) and sGTCs (4.76%, 30.0%, and 55.6%) frequencies rose with age of surgical evaluation. Epileptic spasms, in contrast, were most common in the infant group, with respective frequencies of 33.3%, 16.7%, and 3.7% in the infant, children, and adolescent‐adult groups (Fig. 4).

To analyze the relationship between anatomical dimensions and epilepsy evolution, 33 patients, including 17/30 and 16/28 in the DF‐II and DF‐III groups, respectively, were selected. The mean DEP in the DF‐II group (6.06 ± 3.98 years) was significantly longer than in the DF‐III group (2.78 ± 2.98 years) (*p* = 0.015). The mean volume of both the total HH lesions (1862.5 ± 1191.1 mm^3^) and the intraventricular portion of these lesions (652.5 ± 255.1 mm^3^) in DF‐III patients were significantly increased as compared to the mean total lesion volume in the DF‐II group (275.3 ± 118.8 mm^3^, *p* < 0.05). Pearson correlation analyses of group DF‐III patients revealed that the volume of the intraventricular portion of the HH lesion (*r* = −0.652, *p* = 0.006) rather than total lesion volume (*r* = −0.332, *p* = 0.208), was negatively correlated with DEP. Moreover, the volume of the intraventricular portion of the HH lesions in the DF‐II and DF‐III groups was significantly negatively correlated with DEP (*r* = −0.445, *p* = 0.009). Other topographical measurements, including the depth of invasion in the hypothalamus and the length along the anteroposterior axis of the hypothalamus, were also not significantly correlated with DEP.

### Analyses of predictors of seizure semiology

None of the analyzed predictors were significantly correlated with the onset of autonomic signs, version, dacrystic, blinking, tonic, clonic, spams, or sGTCs. Univariate analyses revealed DF classification to be a predictor of the onset of ictal automatism, with these rates being higher in group DF‐II relative to group DF‐III (DF‐II: 9/17, DF‐III: 2/16, *χ* ^2^ = 6.07, *p* = 0.014). Total HH volume (475.45 ± 581.14 vs. 1329.55 ± 1263.86, *p* = 0.038), the volume of the intraventricular part of the HH lesion (294.55 ± 196.23 mm^3^ vs. 540.00 ± 271.50 mm^3^, *p* = 0.038), MB completeness (Complete: 10/20, incomplete: 2/13, *χ* ^2^ = 4.080, *p* = 0.043) were also significantly associated with the onset of ictal automatism.

Given that DF classification and HH volume were significantly correlated with one another, the total and intraventricular volumes of HH lesions were both excluded from logistic analyses. MB completeness did not differ significantly between patients in the DF‐II and DF‐III subgroups (DF‐II: 13/17, DF‐III: 7/16, *χ* ^2^ = 3.696, *p* = 0.055). As such, a binary logistic regression analysis of DF classification, disease duration, age at surgical evaluation, onset age, gender, and MB completeness was conducted to identify predictors of ictal automatism. This approach revealed that DF classification was a significant predictor of the onset of ictal automatism (*B* = 3.196, *p* = 0.020), which indicates that patients in group DF‐II are more prone to have ictal automatism than those in group DF‐III.

## Discussion

HH lesions are widely accepted to contribute to the development of a distinctive epilepsy syndrome in which patients experience a range of clinical manifestations that evolve over time and are of varying levels of severity. However, there have not been any studies to date of large HH‐related epilepsy patient cohorts that had documented the semiologic spectra or disease evolution dynamics in these patients. The use of high‐resolution whole‐brain volumetric T1 images offers an opportunity to summarize the association between HH anatomical characteristics and epilepsy features. In the present study of a large group of HH patients with epilepsy, we identified several key characteristics of HH‐related epilepsy: (1) GS was the most common seizure type at onset in this patient population (73.1%), with 68.4% of patients ultimately developing additional seizure types following a mean 4.35‐year latency period; (2) Ictal automatism, version, and sGTCs were all common seizure semiologies associated with the evolution of HH‐related epilepsy; (3) the volume of HH lesions, and particularly the volume of the intraventricular portion of these lesions, was closely associated with disease evolution such that smaller lesions were related to more gradual epilepsy evolution; and (4) patients with DF‐I HH lesions generally exhibited atypical seizure manifestations, including later disease onset and lower rates of GS at the time of epilepsy onset. In addition, DF‐II patients were at a higher risk of developing ictal automatism over the course of disease evolution as compared to DF‐III patients.

Gelastic seizures are the characteristic seizure type in HH‐related epilepsy patients, often occurring initially during infancy or early childhood at a high frequency, occurring in clusters in some cases.^3^ In prior studies of HH‐related epilepsy patient cohorts, GS was the initial seizure type in 78.9% (15/19)^4^ or 77.4% (24/31)^5^ of patients, in line with the present results. Stereotactic depth recordings of seizure onset and direct HH stimulation have additionally provided direct evidence for GS ictogenesis,^11^, ^12^, ^13^ while electrical^14^ and ictal SPECT^15^ studies of HH patients have shown that the interface between the HH and the hypothalamus, rather than the entirety of the HH, was associated with seizure incidence.^15^ This is consistent with the finding that intrahypothalamic HH lesions are related to GS onset more often than are parahypothalamic HH cases, as was observed in this study and other past reports.^4^, ^6^, ^16^ As such, efforts to ablate primarily this interface region and to establish a disconnect via endoscopic surgery, stereotactic radiofrequency thermocoagulation,^12^ or laser interstitial thermotherapy^17^, ^18^ is clinically viable. The present results also confirmed that the intraventricular portion of HH lesions was primarily associated with epilepsy disease evolution. Both our results and those of prior studies have shown that small HH lesions generally exhibit reduced epileptic severity and more gradual disease evolution.^19^, ^20^, ^21^ As such, epileptiform activity within the HH has a higher chance of contributing to seizure onset and disease progression when a sessile attachment to the hypothalamus enables it to propagate to the diencephalon and various other cortical structures.^7^, ^22^

Over the course of epilepsy evolution, many other seizure types generally develop in patients following initial GS presentation after a delay,^2^, ^6^ with these seizures being linked to ictal discharges affecting a range of neocortical areas but sparing the HH.^11^, ^23^ This observation is consistent with a model of secondary epileptogenesis as a driver of disease evolution akin to the network hypothesis for focal epilepsy cases. In this patient cohort, the most commonly observed focal seizure behaviors were automatism and version, suggesting the involvement of the mesial temporal lobe and frontal lobe in the spread of these seizures. In both animal and human studies, the anatomic proximity and hypothalamic connections to the MB, fornix, mamillothalamic tract, cingulate gyrus, and amygdala have been explored in detail.^24^, ^25^ SPECT analyses have documented closed functional connectivity between the HH or the associated region of the hypothalamus and the ipsilateral thalamus, particularly the anterior thalamic nucleus, which serves as a relay to the anterior cingulate and limbic circuit, as well as the mediodorsal nucleus, which serves as a relay to the orbitofrontal, anterior cingulate, medial premotor, and parietal cortical regions.^15^, ^23^ Invasive EEG studies have further supported a model in which HH‐related epilepsy is associated with dynamic ictal network organization. Moreover, Kahane et al.^11^ found through SEEG recordings of the HH and the lateral and medial cortices that the mammillo‐thalamo‐cingulate tract can function as a discharge relay from the HH to the cortex.

DF type I HH lesions, also referred to as parahypothalamic hamartomas, can often result in central precocious puberty and, more rarely, can cause late‐onset epilepsy.^26^, ^27^ DF type I HH patients in this study were likely to exhibit non‐GS seizure semiologies including eyelid blinking, automatism, version, and sGTCs. HH invasion depth and the anterior–posterior dimensions of the HH lesion in the hypothalamus offered no predictive value with respect to seizure behaviors. Only patients with DF type II lesions were more likely to develop ictal automatism as compared to patients with DF type III lesions in logistic analyses. No clear mechanism underlying these findings has been established to date. Moreover, no differences in MB involvement were observed among these patient subgroups based on MB completeness, suggesting that such involvement cannot account for the incidence of mesial temporal lobe seizures. Additional research, including diffusion tensor imaging (DTI) and cortico‐cortical evoked potentials, will be necessary to analyze the network mechanisms governing these semiological outcomes.

## Limitations

There are multiple limitations to this study. For one, the age of seizure semiology onset was determined based on clinical interviews and medical records, and as a result, mild GS or other mild events occurring at an early age may have been overlooked. Secondly, seizure events associated with sGTCs incidence were determined based on medical records and could not be confirmed through objective video evidence. Third, neurodevelopmental evaluation and endocrine function were not incorporated into this study.

## Conclusions

In summary, the results of this large cohort study provide insight into seizure semiology and disease evolution in HH‐related epilepsy patients. The most characteristic semiology in these patients was GS, which frequently occurred during early childhood, followed by other seizure semiologies including ictal version and automatism with a mean 4.35‐year duration of onset. DF type II HH patients were also more likely to develop automatism over time. These results also suggest that the size of the intraventricular portion of an HH lesion is a key determinant of disease evolution, which indicates that the intraventricular part of HH is more important when intraoperatively targeting the lesion with ablation techniques, such as radiofrequency thermotherapy and laser interstitial thermotherapy. However, the preliminary results of the present study raise several issues for further research, including how the epilepsy network altered and evolved during DEP and network mechanism for the epileptogenesis of ictal automatism.

## Author Contributions

Conception and design of study—Kai Zhang, Jian‐guo Zhang, Xiaoqiu Shao and Xiu Wang. Acquisition and analysis of data—Xiu Wang, Chang Liu, Zhong Zheng, Wenhan Hu, Chao Zhang, Xiaoli Yang. Drafting a significant portion of the manuscript or figures—Xiu Wang, Chang Liu, Zhong Zheng, Wenhan Hu.

## Funding Information

The present study was supported by National Natural Science Foundation of China (82201603, 82071457, 82271495, 82201600), National Key R&D Program of China (2021YFC2401201) and Capital's Funds for Health Improvement and Research (2022‐1‐1071, 2020‐2‐1076).

## Conflict of Interest Statement

The authors have stated that they had no interests which might be perceived as posing a conflict or bias.

## Supporting information


**Figure S1** Diagram showing the anatomical relationship between the hypothalamus and an HH lesion, including the depth of lateral invasion (A) and the anterior–posterior dimension along the hypothalamus (B). In A, alphabet a represents the line between the fornix (arrow) and the mammillothalamic tract (asterisk) while line b corresponds to the lateral invading depth based on line a. In B, line a corresponds to the vertical line at the midpoint of the mammillary body based on the AC–PC line, while lines b and c correspond to the anterior and posterior dimensions along the hypothalamus.
**Figure S2**. MB involvement was detected based on the completeness of a halo of T1 signal hyperenhancement on MRI images. (A–C) Respectively demonstrate a complete MB without any evidence of the interruption of the halo of signal hyperenhancement in the axial, sagittal, and coronal planes. (D–F) Demonstrate an incomplete MB signal in the axial, sagittal, and coronal planes, respectively. MB, mammillary body.
