# Supplementary material for: Epilepsy in hypothalamic hamartomas: semiology spectrum and predictor analyses of 78 patients
Source: Ann Clin Transl Neurol. 2023 Jun 27;10(8):1365–73. doi: 10.1002/acn3.51827 (PMC10424656; doi:10.1002/acn3.51827)
Supplement: Supplementary file 1 — Figure S1 Diagram showing the anatomical relationship between the hypothalamus and an HH lesion, including the depth of lateral invasion (A) and the anterior–posterior dimension along the hypothalamus (B). In A, alphabet a represents the line between the fornix (arrow) and the mammillothalamic tract (asterisk) while line b corresponds to the lateral invading depth based on line a. In B, line a corresponds to the vertical line at the midpoint of the mammillary body based on the AC–PC line, while lines b and c correspond to the anterior and posterior dimensions along the hypothalamus. Figure S2. MB involvement was detected based on the completeness of a halo of T1 signal hyperenhancement on MRI images. (A–C) Respectively demonstrate a complete MB without any evidence of the interruption of the halo of signal hyperenhancement in the axial, sagittal, and coronal planes. (D–F) Demonstrate an incomplete MB signal in the axial, sagittal, and coronal planes, respectively. MB, mammillary body. [file ACN3-10-1365-s001.docx]

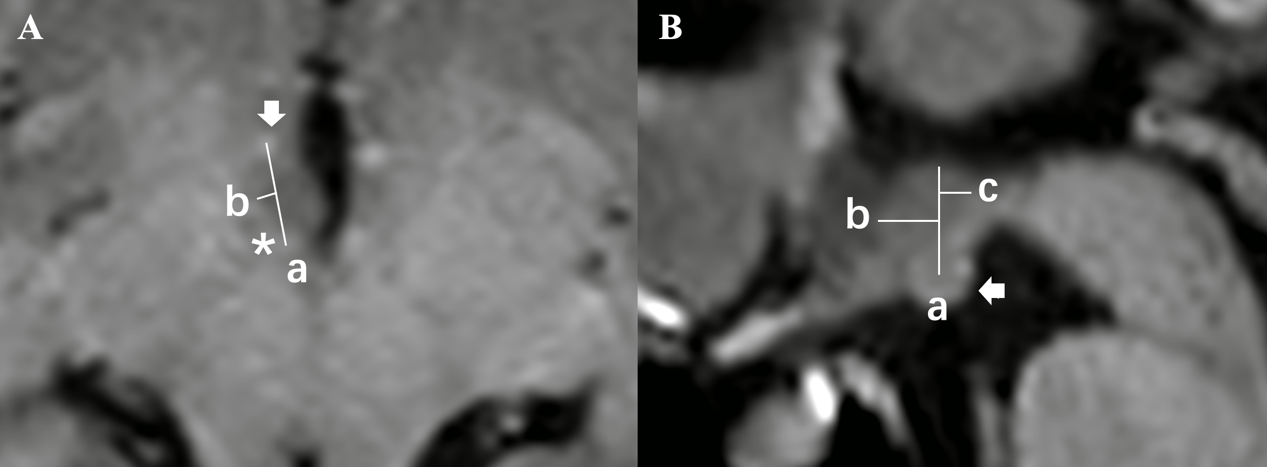


Supplementary Figure-1: Diagram showing the anatomical relationship between the hypothalamus and an HH lesion, including the depth of lateral invasion (A) and the anterior-posterior dimension along the hypothalamus (B). In A, alphabet a represents the line between the fornix (arrow) and the mammillothalamic tract (asterisk) while line b corresponds to the lateral invading depth based on line a. In B, line a corresponds to the vertical line at the midpoint of the mammillary body based on the AC-PC line, while lines b and c correspond to the anterior and posterior dimensions along the hypothalamus.


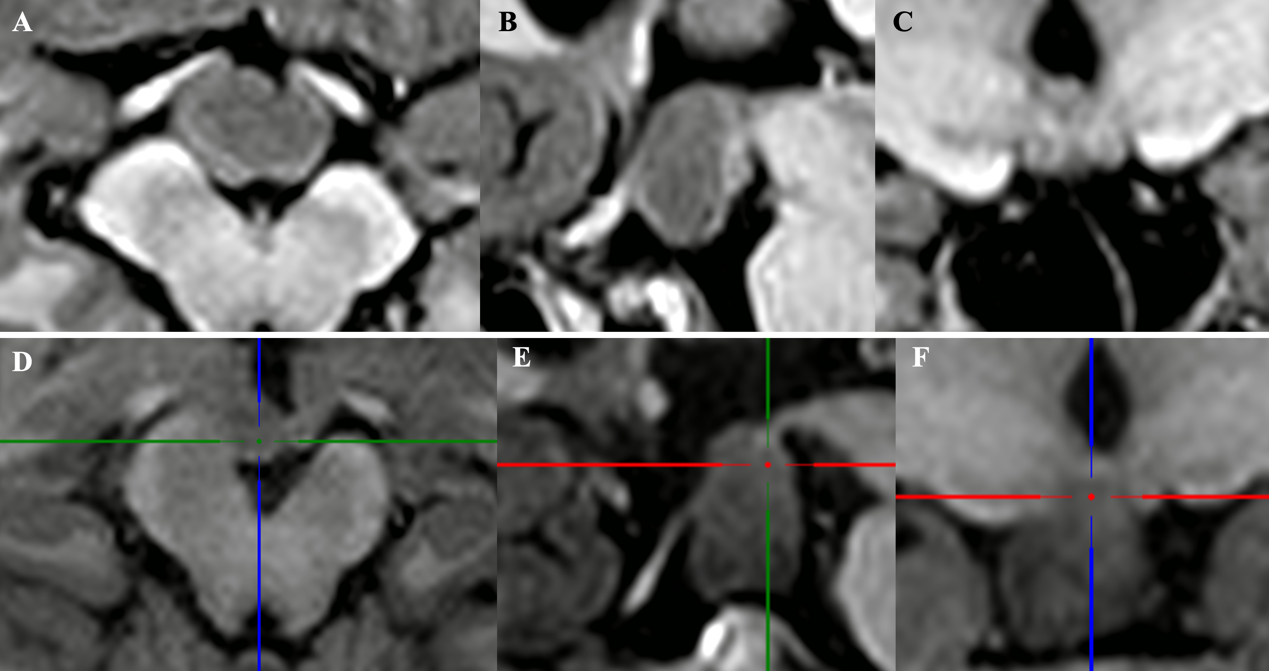


Supplementary Figure-2: MB involvement was detected based on the completeness of a halo of T1 signal hyperenhancement on MRI images. A, B, and C respectively demonstrate a complete MB without any evidence of the interruption of the halo of signal hyperenhancement in the axial, sagittal, and coronal planes. D, E, and F demonstrate an incomplete MB signal in the axial, sagittal, and coronal planes, respectively. MB, mammillary body.
